# Structural basis of *Staphylococcus aureus* Cas9 inhibition by AcrIIA14

**DOI:** 10.1093/nar/gkab487

**Published:** 2021-06-09

**Authors:** Hongnan Liu, Yuwei Zhu, Zebin Lu, Zhiwei Huang

**Affiliations:** Center for Life Sciences, School of Life Science and Technology, Harbin Institute of Technology, 150080 Harbin, China; Center for Life Sciences, School of Life Science and Technology, Harbin Institute of Technology, 150080 Harbin, China; Center for Life Sciences, School of Life Science and Technology, Harbin Institute of Technology, 150080 Harbin, China; Center for Life Sciences, School of Life Science and Technology, Harbin Institute of Technology, 150080 Harbin, China

## Abstract

Bacteriophages have evolved a range of anti-CRISPR proteins (Acrs) to escape the adaptive immune system of prokaryotes, therefore Acrs can be used as switches to regulate gene editing. Herein, we report the crystal structure of a quaternary complex of AcrIIA14 bound SauCas9–sgRNA–dsDNA at 2.22 Å resolution, revealing the molecular basis for AcrIIA14 recognition and inhibition. Our structural and biochemical data analysis suggest that AcrIIA14 binds to a non-conserved region of SauCas9 HNH domain that is distinctly different from AcrIIC1 and AcrIIC3, with no significant effect on sgRNA or dsDNA binding. Further, our structural data shows that the allostery of the HNH domain close to the substrate DNA is sterically prevented by AcrIIA14 binding. In addition, the binding of AcrIIA14 triggers the conformational allostery of the HNH domain and the L1 linker within the SauCas9, driving them to make new interactions with the target-guide heteroduplex, enhancing the inhibitory ability of AcrIIA14. Our research both expands the current understanding of anti-CRISPRs and provides additional culues for the rational use of the CRISPR-Cas system in genome editing and gene regulation.

## INTRODUCTION

The CRISPR-Cas system is an adaptive immune system that bacteria and archaea use to resist the invasion of exogenous nucleic acids. These systems are classified as class 1 and class 2, according to the number of effector proteins involved in immunity (1–3). In class 1 (includes Type I, III and IV), multiple effector proteins are involved in the effector phase. In class 2 (includes Type II, V and VI), only one effector protein is involved in the effector phase and that makes it more widely used in gene editing since its use is more convenient (4,5). Among these, the most widely used is the Type II Cas9 system (6,7). The CRISPR–Cas9 system has two functional components: one recognition (REC) lobe and one nuclease (NUC) lobe (8–11). By targeting a specific genomic sequence complementary to a single guide RNA (sgRNA) and a protospacer adjacent motif (PAM), the HNH domain is primarily responsible for cleaving the complementary strand and the RuvC domain for cleaving non-complementary strands (8–12). The most widely used Type II Cas9 system is *Streptococcus pyogenes* Cas9 (SpyCas9) (5,9). However, its large molecular weight (150kDa) can hamper cell transfection efficiency and therefore affects the application in gene editing (5). Therefore, many researchers are currently looking for more pocket-sized editing proteins that can replace SpyCas9. A good candidate is *Staphylococcus aureus* Cas9 (SauCas9), which is ∼300 amino acids smaller than SpyCas9 (13,14). It was confirmed in previous research that SauCas9 can edit the genome of mammalian cells, potentially with high efficiency and specificity (14).

In the long process of evolution, phages are challenged by bacterial and archaeal CRISPR-Cas systems, and in order to survive they have evolved a counter-measures (15,16). Cas proteins functionality depends on multiple structural rearrangements (17–19) and phages have evolved several strategies to inhibit CRISPR–Cas systems in response to this multistage activation (17,18). Several anti-CRISPR proteins are known to inhibit the CRISPR system and do so through a variety of mechanisms (17,20,21). For example, AcrIIA4 blocks the PAM recognition domain of Cas9 (20), AcrIIC3 promotes Cas9 dimerization (22), AcrVA1 truncates the crRNA of Cas12a (23), AcrVA5 mainly inhibits Cas12a through post-translational acetylation (Ac) (24), AcrIF1 prevents target DNA binding to Cascade (25), AcrIIC1 binds to the catalytic center of the HNH nuclease domain to disable Cas9 (26), AcrIIC2 inhibits Cas9 by preventing sgRNA loading (27) and AcrIE1 binds to Cas3 to block DNA cleavage (28).

In this work, we investigated the mechanism by which AcrIIA14, an anti-CRISPR protein from *Staphylococcus*, inhibits SauCas9 activity (29). AcrIIA14 consists of an N-terminal HTH domain and a C-terminal domain which has previously been reported to inhibit SauCas9 activity *in vivo* and *in vitro* without affecting the binding of SauCas9 protein to sgRNA and target DNA (29) but its specific inhibitory mechanism is still unclear. We further validated the mechanism of this anti-CRISPR protein inhibitting SauCas9 via biochemical assays and structural biology methods. Our results widen our understanding of the diverse anti-CRISPR inhibitory mechanisms and support the use of SauCas9 as a gene editing tool.

## MATERIALS AND METHODS

### Protein expression and purification

The cDNA of full-length SauCas9, SpyCas9, NmeCas9 and AcrIIA14 were synthesized and sub-cloned into a expression vector pGEX-6P-1 (with an N-terminal GST tag). Mutants were constructed using a site-directed mutagenesis kit. All proteins were overexpressed in *E.coli* Rosetta (DE3) (Novagen) cells and were induced with 0.3 mM isopropyl-β-D-1-thiogalactopyranoside (IPTG) at OD600 = 0.6 for 16 h at 18°C. Cells were harvested and resuspended in lysis buffer (25 mM Tris–HCl, pH 8.0, 1500 mM NaCl, 3 mM DTT and 1 mM PMSF), disrupted by sonication, and purified on glutathione sepharose 4B (GS4B) beads (GE Healthcare). Prescission protease was incubated in buffer (25 mM Tris–HCl, pH 8.0, 300 mM NaCl, 3 mM DTT) overnight at 4°C. The cleaved protein was eluted from GS4B resin. For Cas9 proteins, further fractionated by heparin sepharose column and cation exchange chromatography (GE Healthcare). For eluted AcrIIA14ct protein, further fractionated by cation exchange chromatography.

To assemble the SauCas9–sgRNA–AcrIIA14ct–dsDNA complex, SauCas9 (N580A/C946A) protein was incubated with sgRNA (73 nt), AcrIIA14ct and dsDNA at the molar ratio of 1:1.2:5:2. Components were added in the order listed and incubated at room temperature for 5 min and 4°C for 40 min before adding the next component. The complex was applied onto size-exclusion chromatography (Superose 6 increase 10/300 GL, GE Healthcare) with buffer (10 mM Tris–HCl, pH 8.0, 300 mM NaCl, 3 mM DTT) to remove excess sgRNA, dsDNA and AcrIIA14ct. Finally, complexes were concentrated to an *A*_280_ absorbance to 18, as measured by Nanodrop One, before crystallization.

### *In vitro* transcription and purification of sgRNA

*In vitro* transcription with T7 RNA polymerase and template (dsDNA) for sgRNA was generated by PCR. All relevant sequences are listed in Supplementary Table S1.Transcription reactions were performed at 37°C for 4 h in buffer containing 0.1 M HEPES-K, pH 7.9, 12 mM MgCl_2_, 30 mM DTT, 2 mM spermidine, 2 mM each NTP (ATP, UTP, GTP, CTP), 100 μg/ml T7 polymerase and 500 nM transcription template. The sgRNA was purified by gel electrophoresis on a 8% denatured (8 M urea) polyacrylamide gel and followed by running Elutrap system. Finally, the sgRNA was resuspended in DEPC (diethylpyrocarbonate) H_2_O, stored at –80°C.

### Crystallization and structure determination and refinement

The hanging-drop vapor-diffusion method was used for crystal growth. Crystallization conditions were screened on a large scale with mosquito robot and then optimized according to the results of the primary screening. All crystals used for diffraction were obtained by mixing 1 μl SauCas9 complex with 1 μl reservoir solution and incubated at 20°C for 7–14 days. Crystals of the SauCas9–sgRNA–AcrIIA14ct–dsDNA complex were grown from 1.0 M sodium citrate dihydrate, 0.3 M imidazol–HCl, pH 10.0, 0.02 M sodium malonate, pH 7.0.

Before data collection, the crystals were transferred into cryo-protectant buffer (the crystallization buffer containing 20% (w/v) glycerol) and flash-cooled in liquid nitrogen. X-ray diffraction data were collected at beamline BL-17U1 at Shanghai Synchrotron Radiation Facility (SSRF). All images were collected at the wavelength of 0.9791 Å with 0.5° rotation. The diffraction data for SauCas9–sgRNA–AcrIIA14ct–dsDNA was processed with HKL2000. The structure was determined by molecular replacement (MR) with the program PHASER. Each domain of the SauCas9 (PDB 5CZZ) was used as an individual search model for MR. The initial model was improved by several rounds of structural refinement in PHENIX. The final model was validated through MOLPROBITY and PROCHECK. Data collection and structural refinement statistics were listed in supplementary information, Supplementary Table S2. All of the structural figures were prepared using PyMOL.

### *In vitro* DNA cleavage assays

The *in vitro* DNA cleavage assays were performed in 20 μl system containing 500 nM Cas9 RNP complex, 25 nM pUC19 plasmid in the buffer (20 mM HEPES, pH 7.55, 100 mM KCl, 10 mM MgCl_2_, 1 mM DTT and 5% (w/v) glycerol). The pUC19 target DNA plasmid (target DNA with PAM sequence cloned into the pUC19 vector in Ssp1 restriction site) was linearized by EcoR1 digestion before the cleavage reaction. To test AcrIIA14 mediated inhibition of Cas9 to cleave target DNA, pre-incubated of AcrIIA14 with Cas9-sgRNA complex for 5 min at 37°C and molar ratios of Cas9 to AcrIIA14 ranging from 1:0 to 1:4. The cleavage assays were performed at 37°C for 30 min. Reactions were quenched by adding 6× TBE–urea gel loading and ran on 1% agarose gels stained with ethidium bromide for production detection.

### Biolayer interferometry binding assays

All ffinities were measured by biolayer interferometry (BLI) using the ctet RED96 system (FortéBio). All experiments were performed at 20°C with buffer containing 20 mM HEPES, pH 7.55, 100 mM KCl, 10 mM MgCl_2_, 1 mM DTT and 5% (w/v) glycerol. Streptavidin sensors were pre-equilibrated in the buffer for at least 10 min before used in experiments. Biotinylated SauCas9 protein (or SauCas9-sgRNA complex) were loaded onto streptavidin biosensors for 60 s. Binding kinetics were determined from binding data obtained with four or five concentrations of sample. The interference from the biotinylated protein with buffer were analysised as a control. Finally, we calculated the binding constant using Octet data analysis software.

## RESULTS

### Overall structure of AcrIIA14ct bound SauCas9–sgRNA–dsDNA complex

**Figure 1.**
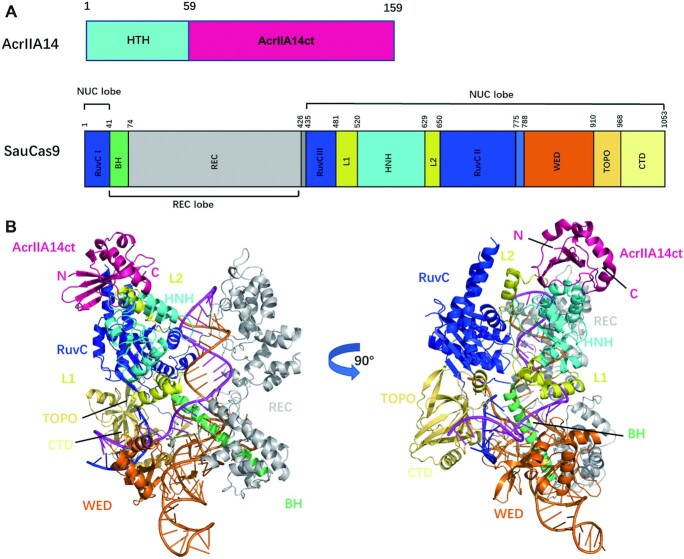
The overall structure of SauCas9–sgRNA–AcrIIA14ct–dsDNA complex. (**A**) Domain organization of AcrIIA14 (29) and SauCas9 (14). (**B**) Overall structure of SauCas9–sgRNA–AcrIIA14ct–dsDNA complex represented as a cartoon. The figure on the right was obtained by a 90° counterclockwise rotation of the figure on the left. Individual SauCas9 domains are colored consistent with the scheme in (A). AcrIIA14ct is colored hotpink.

It was previously reported that three inhibitors of SauCas9, AcrIIA13-AcrIIA15, that share a conserved N-terminal HTH domain which can interact directly with the promoter-proximal sequences of the anti-CRISPR protein locus and plays a role in regulating protein expression, but is not necessary for inhibiting the cleavage activity of SauCas9 (Figure S1A). The C-terminal domain in contrast, here referred to as AcrIIA14ct, is required and spans residues 60–159 (Figure 1A) (29).

In order to elucidate the mechanism of SauCas9 inhibition by AcrIIA14, we determined the crystal structure of SauCas9–sgRNA–AcrIIA14ct–dsDNA quaternary complex at 2.22 Å resolution (Figure 1 and Supplementary Table S2). In the complex, SauCas9 adopts a bi-lobed structure, comprising recognition (REC) and nuclease (NUC) lobes. The channel formed by these two lobes binds the sgRNA–target DNA heteroduplex, as described previously (14). AcrIIA14ct binds to the SauCas9 complex with a 1:1 stoichiometry partially interacting with the L2 linker and making extensive contacts with the HNH domain (Figure 1B), which suggests that this interaction is responsible for SauCas9 inhibition.

### AcrIIA14ct binds HNH domain

**Figure 2.**
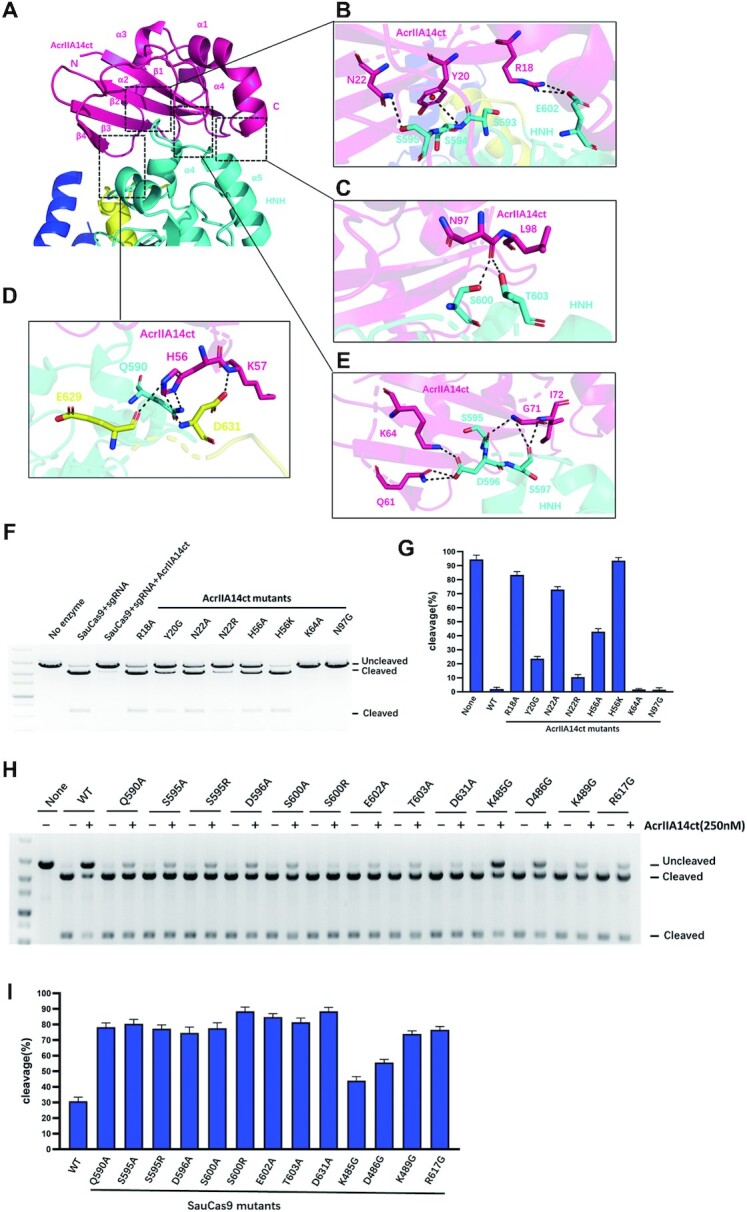
Structural mechanism of AcrIIA14 recognition by SauCas9. (**A**) Cartoon view of the interface between AcrIIA14ct and SauCas9 HNH domain. (B–E) Detailed interactions between SauCas9 and AcrIIA14ct β2 sheet (**B**), loops of α2–β4 (**D**)and β4-α3 (**E**) and C-terminus (**C**). (**F**) *In vitro* DNA cleavage assay using wild-type SauCas9 and AcrIIA14ct mutants at residues involved in HNH recognition. (**G**) Quantitative histogram of substrate cleavage ratio according to (F). (**H**) *In vitro* cleavage assay to verify structural determinants of the AcrIIA14ct-SauCas9 interaction by mixing SauCas9 protein mutants (500 nM) and wild type AcrIIA14ct (250 nM). (**I**) Quantitative histogram of substrate cleavage ratio according to (H). Results shown are representative of three experiments.

AcrIIA14ct consists of a single domain composed of a four-stranded β-sheet with four α-helices positioned along one face (β1β2β3α1α2β4α3α4) (Figure 2A). A DALI search concluded that AcrIIA14ct shares little structural resemblance with currently known proteins, suggesting that it exhibits a novel fold.

Close inspection of the interface between AcrIIA14ct and the HNH domain shows that β2, loop α2–β4, loop β4–α3 and C-terminus of AcrIIA14ct make extensive intermolecular hydrogen bond contacts with α4-α5 of the HNH domain and the loop between the HNH domain and the L2 linker (Figure 2A). Specifically, residues Arg18, Tyr20 and Asn22 from the β2 sheet of AcrIIA14ct make hydrogen bonds with residues Ser593, Ser594, Ser595 and Glu602 from α4-α5 of SauCas9 HNH domain (Figure 2B). In addition, two residues in α2-β4 loop of AcrIIA14ct, His56 and Lys57, make four hydrogen bonds with α4 Gln590 in the HNH domain and Glu629, Asp631 in the loop between the HNH domain and the L1 linker, respectively (Figure 2D). This implies that His56 may be important for AcrIIA14ct recognition of SauCas9. Notably, the protruding β4-α3 loop in AcrIIA14ct is close to the loop α4-α5 in the HNH domain. And residues Gln61, Lys64, Gly71 and Ile72 in AcrIIA14ct interact with residues Ser595, Asp596 and Ser597 of α4-α5 in the HNH domain (Figure 2E). Moreover, the C-terminus of AcrIIA14ct further strengthens the contact through the backbone between residues Asn97 and Leu98 interacting with Ser600 and Thr603 of α5 in the HNH domain (Figure 2C). Consistent with these observations, we found that mutations around the AcrIIA14ct-SauCas9 interface hampered interaction and reducded the effectiveness of AcrIIA14ct as a SauCas9 inhibitor (Figure 2F–I and Supplementary Figure S1C). Notably, AcrIIA14ct mutant H56K significantly reduced the affinity to SauCas9 by four orders of magnitude (*K*_D_ = 58.0 nM versus 5.58 pM for mutant and wild type, respectively) (Supplementary Figure S1C), implying a destabilization of the AcrIIA14ct and Saucas9 complex. Consistent with this, *in vitro* cleavage assays showed the mutant H56K of AcrIIA14ct almost abolished inhibition (Figure 2F and G). Overall, these data indicate that inhibition of SauCas9 is caused by direct interaction between AcrIIA14ct and the HNH domain of SauCas9.

### AcrIIA14ct binding induces SauCas9 allostery

**Figure 3.**
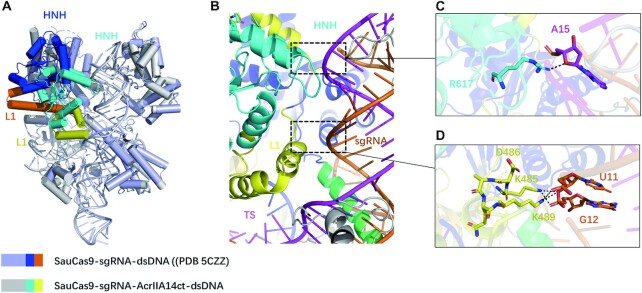
Binding of AcrIIA14 to the HNH domain induces SauCas9 allostery. (**A**) Structural comparison of SauCas9–sgRNA–dsDNA complexes in the bound and unbound states of AcrIIA14ct. The HNH domains in SauCas9–sgRNA–dsDNA and SauCas9-sgRNA-AcrIIA14ct-dsDNA are colored blue and cyan, respectively. The L1 linkers in SauCas9–sgRNA–dsDNA and SauCas9–sgRNA–AcrIIA14ct–dsDNA are colored orange and yellow, respectively. (**B**) Local structure of AcrIIA14 induces SauCas9 allostery. The black boxes represent two new intramolecular connections. Above, shows interaction of SauCas9 HNH domain with the target DNA strand. Below, shows interaction of SauCas9 L1 linker with sgRNA. (**C**) Detailed interaction between the SauCas9 HNH domain with the target DNA strand. (**D**) Detailed interaction between the L1 linker with sgRNA.

To explore how AcrIIA14 binding affects the conformation of SauCas9, we overlapped the structures of SauCas9 when bound to AcrIIA14ct and when bound to dsDNA (PDB 5CZZ). This revealed that AcrIIA14 binding only induces significant conformational changes in the HNH domain and the L1 linker (Figure 3A). In detail, binding of AcrIIA14ct induces HNH domain and L1 linker to approach the target-guide heteroduplex. As a result, the L1 linker becomes two short α helices, whereas the former helix, together with the HNH domain, become closer to the target-guide heteroduplex (Figure 3B). We also found that allostery between these two domains allows the formation of two new interaction surfaces inside the SauCas9 protein complex. First, residue Arg617 in the loop between α5 and α6 of HNH domain hydrogen binds the sugar-phosphate backbone of the target DNA strand (fifteenth nucleotide upstream of the PAM sequence) (Figure 3C). Second, residues Lys485 and Lys489 in the L1 linker interact with the phosphate backbone between the 11th and 12th nucleotides of the sgRNA (Figure 3D). Accordingly, we speculated that these novel intramolecular interactions induced by AcrIIA14ct binding may act to stabilize the inhibitory conformation. To test this, we mutated these three residues separately. As expected, an *in vitro* DNA cleavage assay showed that SauCas9 mutants R617G, K485G and K489G reduced sensitivity to AcrIIA14ct inhibition (Figure 2H and I). In addition, analysis of our structure reveals that residue Asp486 is very important for maintaining the spatial orientation of Lys485 and Lys489 on the L1 linker (Figure 3D). As expected, the inhibitory effect of AcrIIA14ct was also reduced when we introduced the mutation D486A (Figure 2H and I). Taken together, these results suggest that AcrIIA14ct binding induces SauCas9 allostery and that formation of two novel intramolecular interactions further enhance AcrIIA14ct inhibition of SauCas9.

### Mechanism of AcrIIA14 inhibition of SauCas9

**Figure 4.**
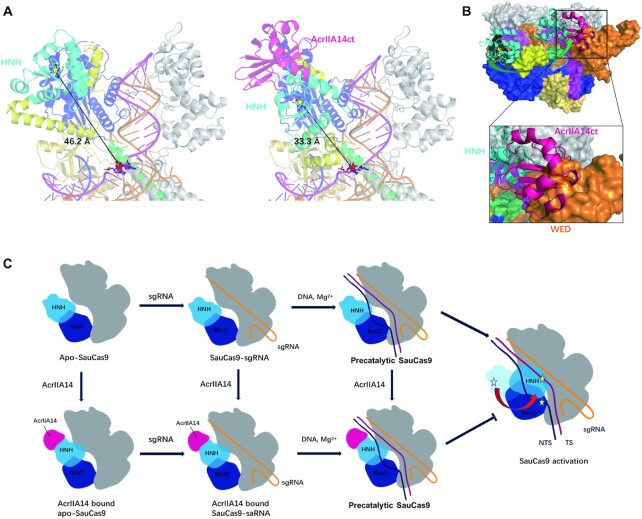
Mechanism of AcrIIA14 inhibition of SauCas9. (**A**) Distance between HNH domain and scissile phosphodiester linkage of SauCas9–sgRNA–dsDNA (PDB: 5CZZ) (left) and SauCas9–sgRNA–AcrIIA14ct–dsDNA (right), with catalytic center of HNH domain (yellow star) and target site (red star) indicated. (**B**) Simulated approximate conformation and location of the HNH domain after SauCas9 activation, based on the previously solved structure of Cas9 in the activated state. The rotation of the HNH domain from the precatalytic to the catalytic state is shown as a green arrow (AcrIIA14ct, hotpink; WED domain of SauCas9, orange). (**C**) Schematic panel of AcrIIA14 inhibition mechanism. The catalytic centers of the HNH domain and the RuvC domain indicated by yellow stars.

In the crystal structure of SauCas9–sgRNA–AcrIIA14ct–dsDNA, the distance between the catalytic center of the HNH domain (D556, H557) and the scissile phosphodiester linkage in the target DNA strand (between the third and fourth nucleotides upstream of the PAM sequence) is ∼33.3 Å (Figure 4A). This suggests that, in this structure, SauCas9 is in the precatalytic state. However, in the SauCas9–sgRNA–dsDNA crystal structure (PDB: 5CZZ) the distance between the HNH domain and the cleavage site is about 46.2 Å (Figure 4A), indicating that the binding of AcrIIA14ct brings the HNH domain ∼13 Å closer to the cleavage site.

According to the previously reported Cas9 activation model, sequential binding of sgRNA and target DNA, the interaction between non-target strand and RuvC domain destroy the fragile stability of the original conformation between the RuvC, L2, L1 and HNH domain, which drives the movement of HNH domain to the cleavage site (30,31). In the AcrIIA14ct bound SauCas9 structure, AcrIIA14ct mainly binds to the region close to the RuvC domain on the HNH domain and makes also some interaction with the anterior segment of the L2 linker (Figure 1B). Therefore, we speculated that the binding of AcrIIA14ct interferes the interaction between the HNH domain and the RuvC domain, L1 and L2 linker. This leads to the proximity of HNH domain to the target-guide heteroduplex, just like the movement of HNH domain during activation. This large movement further facilitates the allostery of the L1 linker.

As described above, during Cas9 activation the HNH domain undergoes a large-scale rotation and translocation. Based on the previously solved structure of Cas9 in the activated state (11,30), we simulated the approximate conformation and location of the HNH domain after SauCas9 activation (Figure 4B). In this simulated structure, there is a steric clash between AcrIIA14ct and the WED domain, indicating that AcrIIA14ct binding to HNH domain sterically prevents the HNH domain from transitioning to the catalytic state. It is known that the L1 linker plays a very critical role in the movement of the HNH domain to the cleavage site (11,14), therefore the change in the position and conformation of the L1 linker caused by the binding of AcrIIA14ct also prevents activation of SauCas9.

From the above structural analysis, AcrIIA14ct binding leads to the formation of two new interactions between the HNH domain and L1 linker and the target-guide heteroduplex in the SauCas9 complex. Mutating the residues that mediate these interactions reduced the effectiveness of AcrIIA14ct (Figure 2H and I). Therefore, we speculate that these novel interactions stabilize the inhibitory state of SauCas9, further improving the inhibitory activity of AcrIIA14ct. This is consistent with its robust inhibitory capacity (Supplementary Figure S1A). Of course, more direct assays are needed to verify these conclusions. Together, our structural and biochemical results demonstrate that AcrIIA14ct inhibits SauCas9 activity by directly binding to its HNH domain and preventing its movement towards the cleavage site (Figure 4B and C). In addition, we speculate that the inhibitory activity of AcrIIA14 is enhanced by stabilizing a SauCas9 protein inhibited state.

### Comparison of AcrIIA14 with AcrIIC1 and AcrIIC3

**Figure 5.**
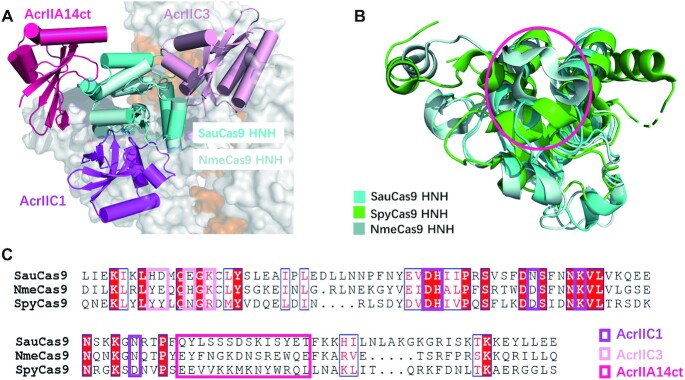
Comparison of AcrIIA14 with AcrIIC1 and AcrIIC3. (**A**) Structural comparison of SauCas9–sgRNA–AcrIIA14ct–dsDNA, NmeCas9 HNH domain-AcrIIC1 (PDB 5VGB) and NmeCas9–sgRNA–AcrIIC3 (PDB: 6JE9) complexes, showing that the three Acrs bind different regions of the HNH domain. (**B**) Superimposition of the HNH domains of SauCas9 (cyan), SpyCas9 (green) (PDB 4OO8) and NmeCas9 (palecyan) (PDB: 6JDQ). The binding region of AcrIIA14 is shown as a red circle. (**C**) Multiple sequence alignment of the HNH domains of SauCas9, NmeCas9 and SpyCas9. Residues involved in the interaction with different Acrs are surrounded by boxes of different colors.

Like AcrIIA14, AcrIIC1 and AcrIIC3 prevent Cas9 protein from cleaving target DNA via direct binding to the HNH domain, whereas their action does not interfere with sgRNA and target DNA binding (11,26). To compare their Cas9 inhibition mechanisms, we superimposed structures of SauCas9–sgRNA–AcrIIA14ct–dsDNA, NmeCas9 HNH domain–AcrIIC1 and NmeCas9–sgRNA–AcrIIC3 (Figure 5A). Three Acr proteins display distinct overall architectures and bind at different positions of the HNH domain. For example, the β sheet of AcrIIC1 inserts into the HNH domain active groove (Figure 5A) preventing the catalytic center from accessing the target DNA and likely excluding the divalent cation. In contrast, AcrIIC3 binds to a surface of the HNH domain close to the target-guide heteroduplex (Figure 5A). According to a previous structure of Nme1Cas9–sgRNA–AcrIIC3, two AcrIIC3 proteins aggregation two Nme1Cas9 proteins together, each AcrIIC3 protein interacts with the HNH domain of one Nme1Cas9 and the REC2 domain of the other. Thus, the two AcrIIC3 proteins lock the position of the HNH domain by bridging the two Cas9 proteins in a quaternary complex, hindering rotation towards the target DNA strand(Supplementary Figure S2A).

In contrast to AcrIIC3, AcrIIA14 binds on a surface of the HNH domain far from the nucleic acid heteroduplex, interacting with the protrusion formed by the two α helices of the HNH domain (Figure 5A). This sterically prevents the HNH domain from moving to the cleavage site of target DNA. In addition, it drives a significant change in the conformation of SauCas9 HNH domain and L1 linker, enhancing its inhibitory ability, as described above. However, structural superposition of Cas9 complexes bound to AcrIIC3 or sgRNA show nearly identical structures of NmeCas9–sgRNA in these two complexes (Supplementary Figure S2B), indicating that AcrIIC3 binding does not induce a conformational change of NmeCas9 and sgRNA. Since the structure of intact Cas9 protein bound to AcrIIC1 is not available, it is unclear whether AcrIIC1 binding can also induce changes in the Cas9 protein. Taken together, comparison of the structures of AcrIIA14, AcrIIC1 and AcrIIC3 bound Cas9 complex, shows that AcrIIA14 binds to a different surface of the HNH domain, enhancing its inhibitory ability by inducing Cas9 protein allostery, in contrast to AcrIIC1 and AcrIIC3.

Further, we also compared the surfaces of the HNH domain in the three Type II Cas9 proteins, SauCas9, NmeCas9 and SpyCas9, recognized by the three Acr proteins. Sequence conservation analysis shows that only the residues bound by AcrIIC1 are highly conserved (Figure 5C). Structural comparison of these three HNH domains also show that the catalytic center bound by AcrIIC1 is conserved, whereas the regions bound by AcrIIA14 and AcrIIC3 are not (Figure 5B and Supplementary Figure S2C). This is consistent with our *in vitro* cleavage assays (Supplementary Figure S1A and S1B) and previous reports that show that AcrIIA14 and AcrIIC3 specifically inhibit SauCas9 and NmeCas9, respectively, whereas AcrIIC1 can inhibit diverse Cas9 orthologs. In summary, in contrast with AcrIIC1 and AcrIIC3, AcrIIA14 identifies a novel non-conserved HNH surface and inhibits specifically SauCas9 activity.

## DISCUSSION

In the present work, we have solved the crystal structure of the quaternary complex formed by SauCas9–sgRNA–AcrIIA14ct–dsDNA at 2.22 Å resolution. This high-resolution structure allows us to clearly describe the mechanism of SauCas9 inhibition by AcrIIA14. Our results indicate that AcrIIA14ct binds to SauCas9 HNH domain. This binding sterically prevents the movement of the HNH domain toward the scissile phosphodiester linkage in the target DNA strand (Figure 4B and C). At the same time, it positions the HNH domain and the L1 linker closer to the target-guide heteroduplex, further improving its inhibitory ability. In contrast to AcrIIC1 and AcrIIC3, AcrIIA14ct binds to a novel non-conserved region of the HNH domain, which results in AcrIIA14ct inhibition being specific to SauCas9. The fact that diverse Acr proteins can target different surfaces of the HNH domain suggests that HNH allostery is very important during Cas9 protein activation. Additionally, it may be a mechanism used by phages to evade bacterial anti-anti-CRISPR strategies in this ongoing arms race.

AcrIIA14ct is able to bind SauCas9 apo, sgRNA-bound or DNA-bound precatalytic SauCas9 (Figure 4C). This is consistent with the fact that the HNH domain is not involved in sgRNA or target DNA binding (9,31,32). The ability of AcrIIA14 to bind Cas9 protein in multiple states may enhance the inhibition of bacterial CRISPR systems during phage invasion. We also found that the affinity of SauCas9 to AcrIIA14ct (*K*_D_ = 5.58 pM) is significantly higher than to sgRNA (*K*_D_ = 473 pM) or to target DNA (535 pM) (Supplementary Figure S1C). This allows AcrIIA14 to inhibit Cas9 protein before its activation by sgRNA and target DNA, ensuring that phages win the arms race with bacteria. In a wider context, our studies expand the understanding of the diverse anti-CRISPR inhibitory mechanisms.

## DATA AVAILABILITY

The atomic coordinates of the crystal structure of SauCas9-sgRNA-AcrIIA14ct-dsDNA have been deposited in the Protein Data Bank (PDB ID 7EL1).

## Supplementary Material

gkab487_Supplemental_Files
