# Supplementary material for: Structural basis of Staphylococcus aureus Cas9 inhibition by AcrIIA14
Source: Nucleic Acids Res. 2021 Jun 9;49(11):6587–95. doi: 10.1093/nar/gkab487 (PMC8216286; doi:10.1093/nar/gkab487)
Supplement: gkab487_Supplemental_Files [file gkab487_supplemental_files.zip › Supplementary Material.pdf]

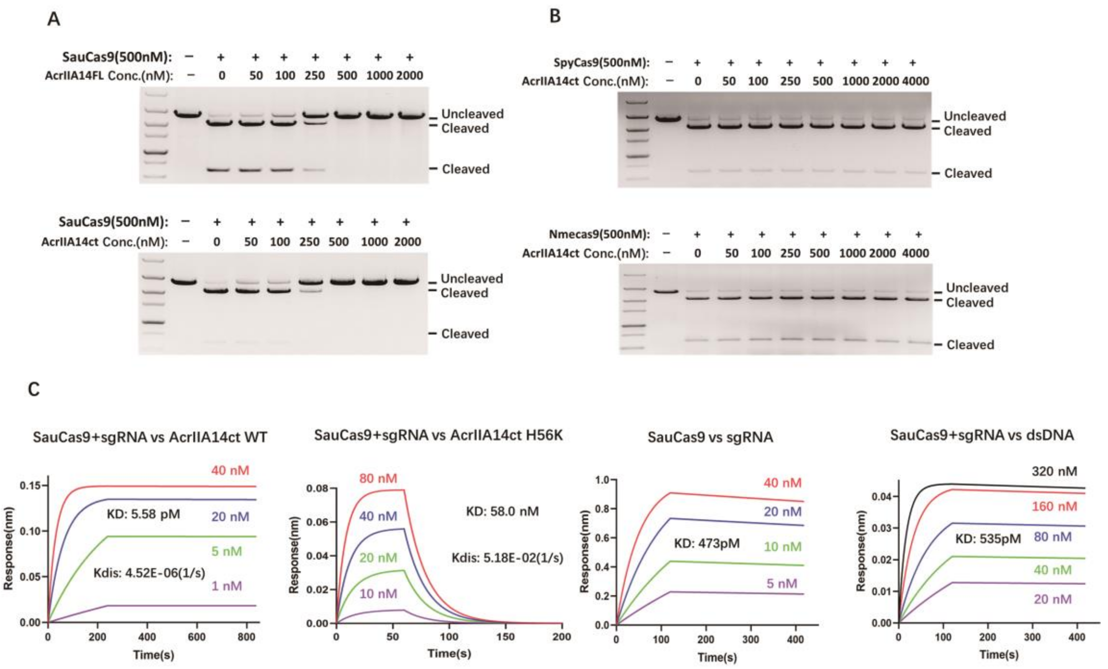

Figure S1. *In vitro* cleavage and binding kinetics assays. (**A**) *In vitro* cleavage assays were performed to compare the SauCas9 inhibition by full-length AcrIIA14 (upper panel) or its C-terminal domain, AcrIIA14ct (lower panel). The molar concentrations of anti-CRISPR protein and SauCas9 are shown at the top of each lane. The reactions were quenched by addition of 6× TBE-urea gel loading and were run on 1% agarose gels stained with ethidium bromide. (**B**) *In vitro* cleavage assays performed to test SpyCas9 and NmeCas9 inhibition by AcrIIA14ct. (**C**) Binding kinetics of (from left to right) SauCas9-sgRNA to wild type AcrIIA14ct or its mutant H56K, SauCas9 to sgRNA and SauCas9-sgRNA to dsDNA. These were measured by biolayer interferometry (BLI) using the ctet RED96 system (FortéBio). In all binding kinetic assays, $R^2 > 0.95$. The results shown are representative of three experiments.

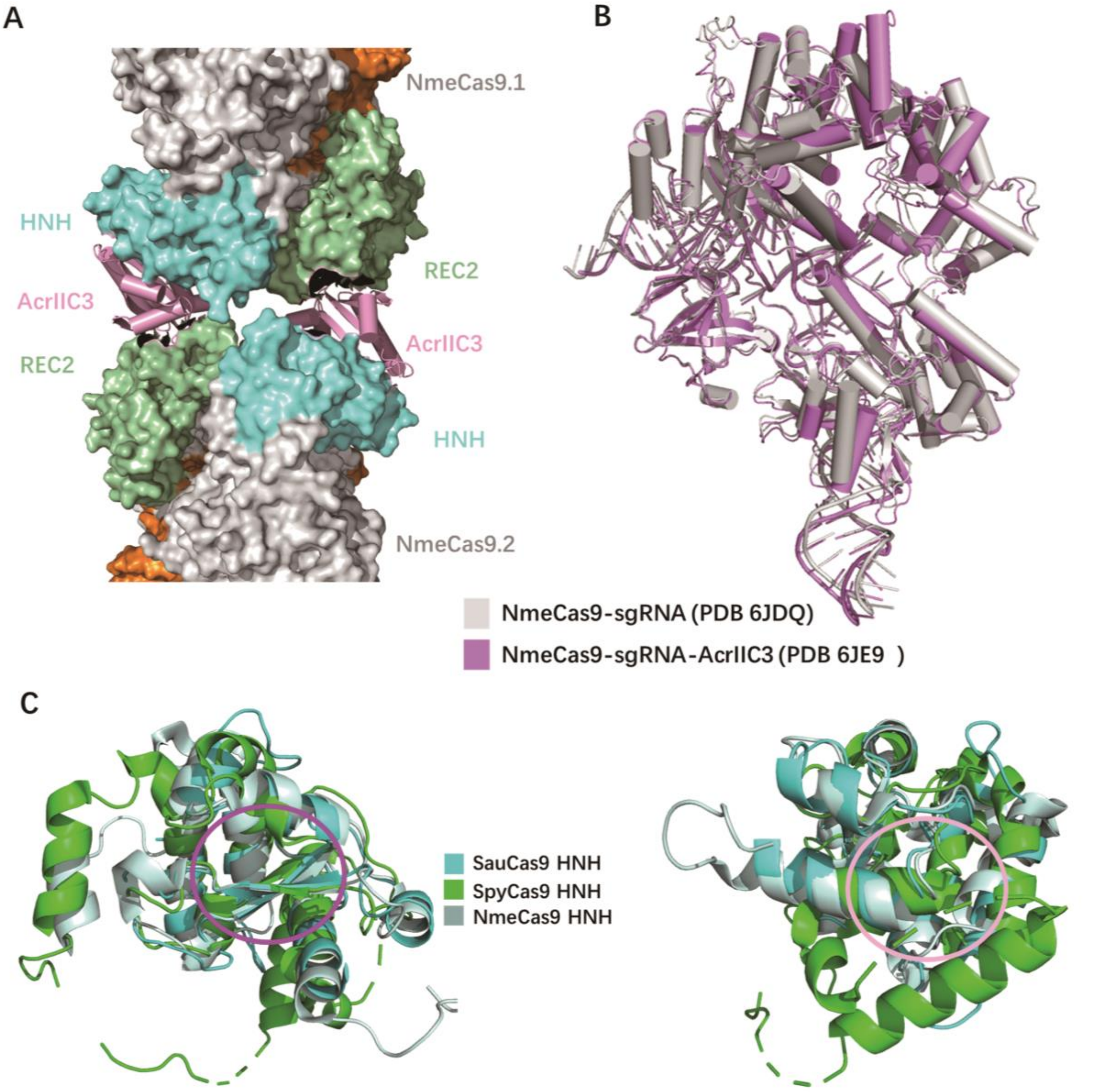

Figure S2. Structural comparison of AcrIIA14 with AcrIIC1 and AcrIIC3. (**A**) Structure of NmeCas9-sgRNA-AcrIIC3 (PDB 6JE9). (**B**) Superimposition of NmeCas9-sgRNA-AcrIIC3 (PDB 6JE9) and NmeCas9-sgRNA (PDB 6JDQ). (**C**) Superimposition of the HNH domains of SauCas9 (cyan), SpyCas9 (green) (PDB 4OO8) and NmeCas9 (palecyan) (PDB 6JDQ), with AcrIIC1 binding region (purple, left) and AcrIIC3 binding region (yellow, right).

Table S1. Oligonucleotide Sequences Used in This Study

	Sequence (5' to 3')
SauCas9 sgRNA(73nt) (Crystallography)	GGAAAUUAGGUGCGCUUGGCGUUUUAGUACUCUGGAAACAGA AUCUACUAAAACAAGGCAAAAUGCCGUGUUU
SauCas9 sgRNA(101nt) (cleavage assay)	GGAAAUUAGGUGCGCUUGGCGUUUUAGUACUCUGGAAACAGA AUCUACUAAAACAAGGCAAAAUGCCGUGUUUAUCUCGUCAACU UGUUGGCGAGAUUUUU
SpyCas9 sgRNA(103nt) (cleavage assay)	GGCGCAUAAAGAUGAGACGCGUUUUAGAGCUAGAAAUAGCAAGU UAAAAUAAGGCUAGUCCGUUAUCAACUUGAAAAAGUGGCACCGA GUCGGUGCUUUUUUU
NmeCas9 sgRNA(132nt) (cleavage assay)	GGUCACUCUGCUAUUUAACUUUACGUUGUAGCUCCCUUUCUCGAA AGAGAACCGUUGCUACAAUAAGGCCGUCUGAAAAGAUGUGCCGCA ACGCUCUGCCCCUUAAAGCUUCUGCUUUAACGGGCUUUUUUU
SauCas9 target DNA (Crystallography)	CTATTCAAGCCAAGCGCACCTAATTTCC
SauCas9 non-target DNA (Crystallography)	TTGAATAG
SauCas9 target DNA cloned into pUC19	GGAAATTAGGTGCGCTTGGCTTGAATAG
SpyCas9 target DNA cloned into pUC19	GGCGCATAAAGATGAGACGCTGGCGATTAG
NmeCas9 target DNA cloned into pUC19	GGTCACTCTGCTATTTAACTTTACATATGATTTTA

Table S2. Data collection and refinement statistics

	SauCas9-sgRNA-dsDNA-AcrIIA14
Data collection	
Beam Line	BL17U, SSRF
Space Group	C121
Unit Cell Parameters	
a, b, c (Å)	330.59, 105.22, 68.55
α, β, γ (˚)	90.00, 92.71, 90.00
Wavelength(Å)	0.9791
Resolution limits(Å)	68.47-2.22(2.34-2.22)
No. of unique reflections	116344(16935)
Completeness (%)	99.9(100.0)
Redundancy	6.4(5.5)
R_{merge} (%) [a]	5.2(71.4)
Mean I/σ(I)	17.0(2.7)
Refinement	
Resolution limits(Å)	50.00-2.22
No. reflections	116174
R_{work}(%) [b]/R_{free}(%) [c]	21.32/24.23
R.m.s.d for bonds (Å)	0.009
R.m.s.d for angles (˚)	1.149
Averaged B factor of the structure	54.95
No.of non-hydrogen protein atoms	11637
Ramachandran plot (%)	
Preferred region	96.53
Allowed region	3.38
Outliers	0.09

Highest-resolution shell is shown in parenthesis.

[a] $R_{merge} = \sum|I_i - \langle I \rangle|/\sum|I|$, where I_i is the intensity of an individual reflection and $\langle I \rangle$ is the average intensity of that reflection.

[b] $R_{work} = \sum||F_o| - |F_c||/\sum|F_o|$, where F_o and F_c are the observed and calculated structure factors for reflections, respectively.

[c] R_{free} was calculated as R_{work} using the 5% of reflections that were selected randomly and omitted from refinement.
