# Supplementary figures and images for: Structural basis of Staphylococcus aureus Cas9 inhibition by AcrIIA14
Source: Nucleic Acids Res. 2021 Jun 9;49(11):6587–95. doi: 10.1093/nar/gkab487 (PMC8216286; doi:10.1093/nar/gkab487)

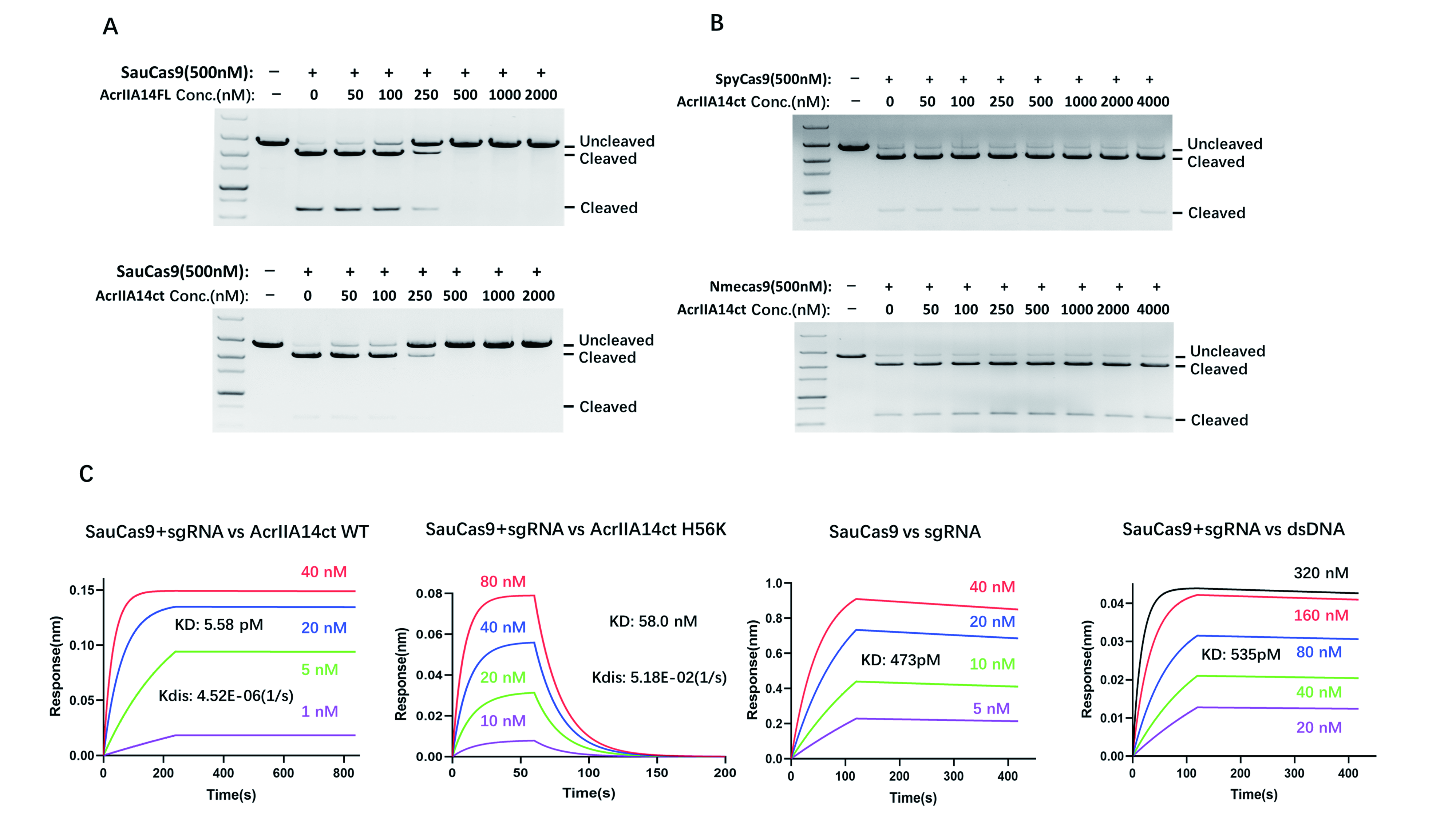

Supplement: gkab487_Supplemental_Files [file gkab487_supplemental_files.zip › Figure S1.tif]

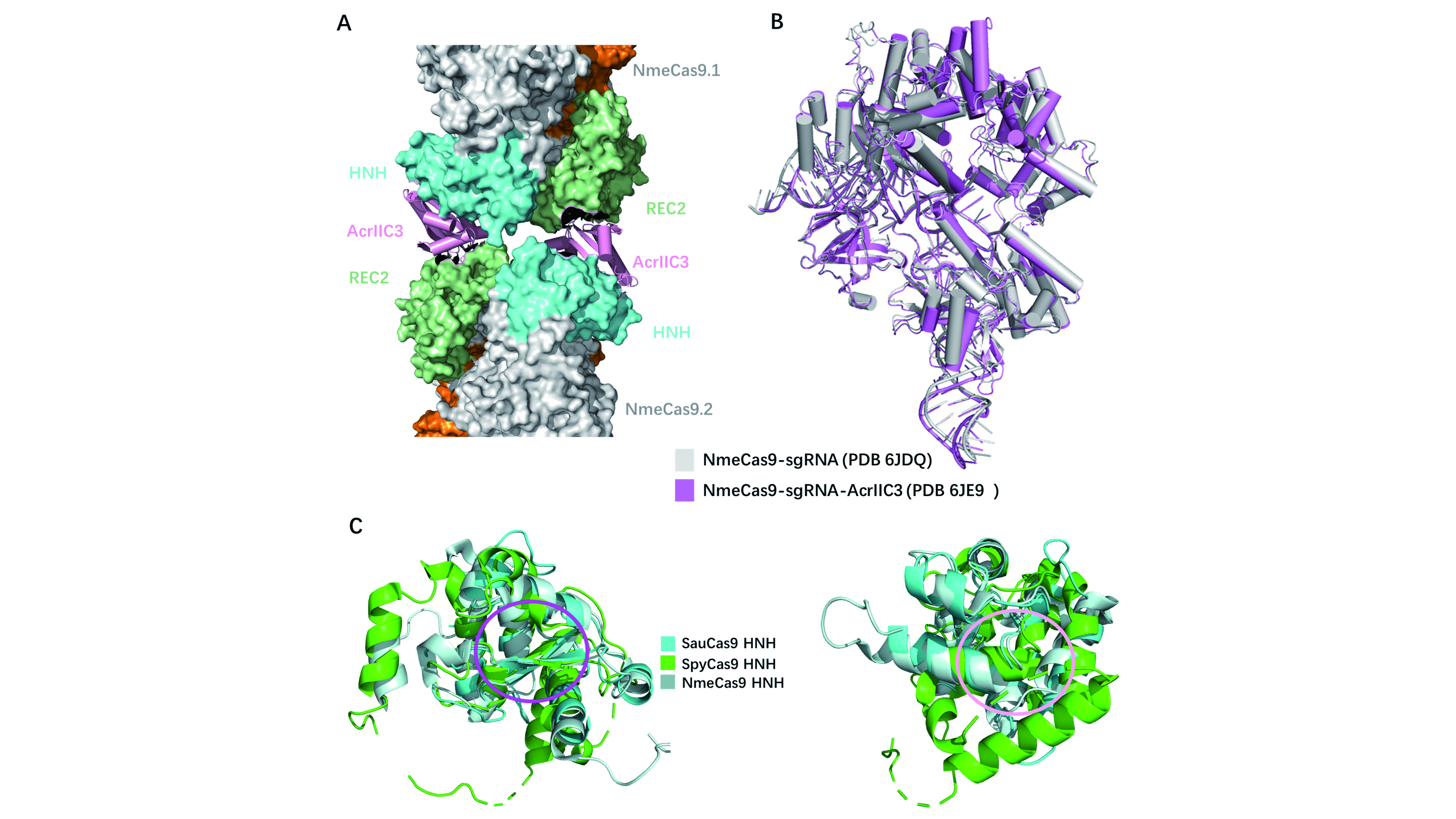

Supplement: gkab487_Supplemental_Files [file gkab487_supplemental_files.zip › Figure S2.tif]
